# Winter Green Manure Decreases Subsoil Nitrate Accumulation and Increases N Use Efficiencies of Maize Production in North China Plain

**DOI:** 10.3390/plants12020311

**Published:** 2023-01-09

**Authors:** Zonghui Hu, Qiu Zhao, Xinjian Zhang, Xiaoguang Ning, Hao Liang, Weidong Cao

**Affiliations:** 1 College of Agricultural Science and Engineering, Hohai University, Nanjing 210098, China; 2 Tianjin Academy of Agricultural Sciences, Tianjin 300192, China; 3 Key Laboratory of Plant Nutrition and Fertilizer, Ministry of Agriculture and Rural Affairs/Institute of Agricultural Resources and Regional Planning, Chinese Academy of Agricultural Sciences, Beijing 100081, China

**Keywords:** nitrogen use efficiency, North China Plain, spring maize, winter green manure, yield

## Abstract

Planting a deep-rooted green manure (GM) (more than 1.0 m depth) greatly improves soil fertility and reduces the loss of nutrients. However, few studies have examined the response of soil nitrogen (N) distribution in the soil profile and subsoil N recovery to the long-term planting and incorporation of deep-rooted GM. Based on a 12-year (2009–2021) experiment of spring maize-winter GMs rotation in the North China Plain (NCP), this study investigated the effects of different GMs that were planted over the winter, including ryegrass (RrG, *Lolium* L.) (>1.0 m), Orychophragmus violaceus (OrV, *Orychophragmus violaceus* L.) (>0.8 m), and hairy vetch (VvR, *Vicia villosa* Roth.) (>1.0 m), on the spring maize yield, N distribution in the deep soil profile, N use efficiencies, functional gene abundances involving soil nitrification–denitrification processes and N_2_O production. Compared with the winter fallow, the maize yield significantly increased by 11.6% after 10 years of green manuring, and water storage in 0–200 cm soil profile significantly increased by 5.0–17.1% at maize seedling stage. The total N content in the soil layer at 0–90 cm increased by 15.8–19.7%, while the nitrate content in the deep soil layer (80–120 cm) decreased by 17.8–39.6%. Planting GM significantly increased the N recovery rate (10.4–32.7%) and fertilizer N partial productivity (4.6–13.3%). Additionally, the topsoil N functional genes (ammonia-oxidizing archaea *amoA,* ammonia-oxidizing bacterial *amoA, nirS, nirK*) significantly decreased without increasing N_2_O production potential. These results indicated that long-term planting of the deep-rooted GM effectively reduce the accumulation of nitrates in the deep soil and improve the crop yield and N use efficiencies, demonstrating a great value in green manuring to improve the fertility of the soil, increase the crop yield, and reduce the risk of N loss in NCP.

## 1. Introduction

Intensification of crop production is widely adopted in many regions in order to meet the increasing global demands for grain [1]. The North China Plain (NCP) is an important grain-producing area where the cropland has a high intensification of the double cropping winter wheat–summer maize production system [2]. The average nitrogen (N) application rate in this system is 550–660 kg ha^−1^ yr^−1^. Moreover, soil residual N and nitrate leaching accounted for approximately 50% of the total input of N, whereas the crop N uptake accounted for only 27% [3]. Studies have revealed that water pollution from nitrates is often associated with an increased use of N in fertilizer [4], and the excess N in fertilizer mainly accumulates in the deep soil layer (90–210 cm), posing a major threat of groundwater contamination [5]. A large accumulation of mineral N in the subsoil (>40 cm) was found in many sites due to improper management of water and fertilizer, resulting in a high risk of pollution in the groundwater in NCP [6].

In addition, farming NCP has extracted groundwater excessively over the past 30 years due to high irrigation requirements for crop production and the uneven distribution of seasonal precipitation. For the winter wheat–summer maize double cropping system, a large amount of irrigation water was extracted from the groundwater for production of winter wheat, contributing to a drop in the groundwater level at a rate of 1–2 m per year [7]. This causes a tremendous depression funnel area to emerge, resulting in damage to the ecological environment [8]. To develop usable and sustainable groundwater with agricultural production, a few croplands in the NCP implemented conservation patterns in winter, converting the traditional double winter wheat–summer maize system into a monoculture spring maize system, which provides the needed opportunity for the development of winter GM in this region [9].

During the growth of GM, a leguminous GM fixes N_2_ in the atmosphere, and the non-leguminous GM can uptake and utilize mineral N in the soil profile to form biomass N. After the incorporation of GM, biomass N was added into soil and provided the N nutrient for subsequent crops [10]. However, large amounts of mineral N can suppress N fixation by 50% in soil treated with long-term fertilizers [11]. Numerous studies have shown that long-term planting and the utilization of GM can increase soil organic matter by 15.5% [12], improve soil structure [13], reduce nitrate leaching (approximately 50%) [14], and increase the crop yield by 4.3% [15]. In Sweden, it was found that planting GM for six consecutive years could reduce the accumulation of mineral N in the soil and reduce the loss of N [16]. GM can also enhance the uptake of N in crops by 18.4% to 62.2% [17]. Specifically, *Orychophragmus violaceus*, commonly called Chinese violet cress, belongs to the cruciferous family and is native to central China. Zhang et al. [18] reported that *Orychophragmus violaceus*–maize rotation improved soil chemical properties and plant nutrient uptake. Based on a meta-analysis, Fan et al. [19] showed that GM significantly increased the crop yield by 9.7% compared with the fallow season. Deep-rooted GM promotes nutrient cycling and redistributes N in soil profile by the interaction of deep-root system and subsoil [20]. However, previous studies mainly concentrated on analyzing the properties of topsoil layer [21]. The effect of the long-term GMs application on subsoil N utilization is still unclear.

Furthermore, GMs also play important role in regulating soil nitrification and denitrification processes, thereby impacts soil N loss and N_2_O emission [22]. Nitrification is a process that NH_4_^+^-N is oxidized to NO_3_^−^-N under the action of ammonia-oxidizing microorganisms, which mainly related to archaea-*amoA* (AOA-*amoA*, ammonia oxidizing archaea) and bacteria-*amoA* (AOB-*amoA*, ammonia oxidizing bacteria) [23]. Denitrification mainly consists of two key steps, including the conversion of NO_3_^−^-N to N gaseous (NO, N_2_O, and N_2_), which involves *narG*, *nirS*, *nirK,* and *nosZ* genes [24]. There were many studies reported the change of functional genes after incorporating GM. However, most focused on N cycling processes on the topsoil layer [25,26,27]. Few studies had simultaneously evaluated the differences of water and nitrogen distribution and nitrogen functional genes throughout the whole soil profile after long-term green manuring.

Therefore, the main objectives of study were to (i) analyze the response of N distribution in the soil profile (0–200 cm) for long-term planting and the utilization of GM, (ii) evaluate the long-term effects of different GMs on the spring maize yield and N use efficiencies; (iii) determine how long-term green manuring affects soil N cycles which related to potential N_2_O production.

## 2. Results

### 2.1. Crop Yield and N Use Efficiencies

There was no significant difference in spring maize yield among different treatments in the first nine years (Table 1). The average yields (2013–2020) of the FW, RrG, VvR, and OrV treatments were 8126, 8691, 8340, and 8269 kg ha^−1^, respectively. Interestingly, the spring maize yield of RrG, VvR, and OrV treatments in 2019 (and 2020) increased by 6.3% (and 14.6%), 11.7% (and 1.1%), and 16.6% (and 11.5%), respectively, compared to the FW treatment, indicating that long-term winter GM can improve the yield of spring maize.

In addition, different winter GMs had a significant impact on crop N uptake and N use efficiencies. Grain N, straw N, NRE, and NPFP of maize ranged from 93.1 to 129.3 kg N ha^−1^, 62.6 to 74.1 kg N ha^−1^, 35.9 to 41.9 kg kg^−1^, and 44.4 to 58.9% under different treatments, respectively (Table 2). Crop N uptake, NRE, and NPFP in the RrG, OrV, and VvR were significantly higher than those in the FW treatment, and they increased by 6.1–19.5%, 10.4–32.7%, and 4.6–13.3%, respectively, indicating that GM improves the crop N uptake and N use efficiencies. Among them, the VvR had the highest crop N uptake and NRE, and the OrV had the highest NPFP.

### 2.2. Soil Water and pH in the Soil Profile

**Figure 1:**
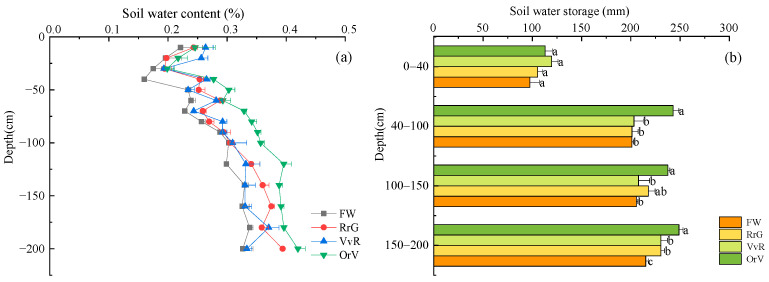
Effects of long-term green manuring on soil water content (**a**) and soil water storage (**b**) in the soil profile (0–200 cm). Error bars are based on standard deviation of three replicates. Different lowercase letters indicate significant differences at the *p*  <  0.05 level within the same treatment group.

Long-term winter green manuring had a significant influence on the soil water distribution in the soil profile (Figure 1a). The soil water content ranged from 15.9% to 41.9% across the soil profile. Compared with FW, GMs increased soil water content of the soil layer at 0–200 cm, especially in the deep soil water content (>40 cm) where the OrV treatment had the highest water content (Figure 1a). Soil water storage at 40–100 cm and 100–150 cm showed similar results. Compared with FW, the water storage of the OrV treatment increased significantly by 20.8% and 15.5%, respectively, and there was no significant difference between the RrG (and VvR) and FW treatments (Figure 1b). The soil water storage of the RrG, VvR, and OrV treatments in the soil layers at 150–200 cm increased significantly by 7.2%, 7.1%, and 15.7%, respectively, compared to the FW treatment (Figure 1b). The soil pH of the GM treatments at a depth of 0–10 cm was lower than the FW treatment (9.07) with an average value of 8.96 (Figure S2a). Long-term planting of different GMs had no significant effect on the pH of the subsoil layers, except for the surface layer.

### 2.3. Nitrogen Distribution in the Soil Profile

**Figure 2:**
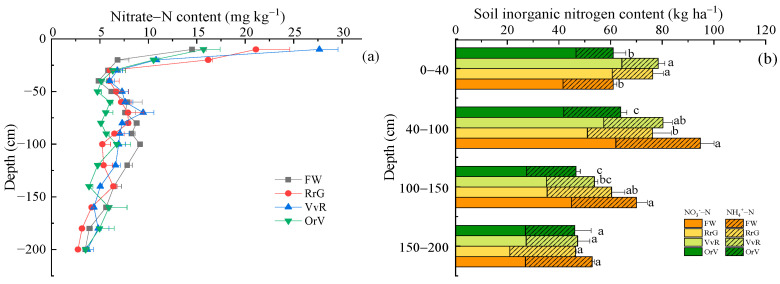
Effects of long-term green manuring on soil nitrate distribution (**a**) and inorganic N accumulation (**b**) in the soil profile (0–200 cm). Error bars are based on standard deviation of three replicates. Different lowercase letters indicate significant differences at the *p*  <  0.05 level within the same treatment group.

After long-term winter planting and the utilization of the GM, the soil nitrate content showed an overall “S”-shaped change tendency during the spring maize seedlings stage (Figure 2a). The incorporation of GM significantly increased the nitrate content in the topsoil (0–20 cm). Compared with the FW treatment, the nitrate content in the RrG, VvR, and OrV treatments increased significantly by 59.3%, 66.5%, and 19.8%, respectively. Additionally, the opposite trend was shown in the soil layer at a depth of 80–120 cm. The nitrate content of the GM treatments was lower than that of the FW treatment, the average soil nitrate content of the FW treatment was 8.09 mg N kg^−1^, and the average soil nitrate N content decreased most significantly in the OrV treatment at 34.6%. The NH_4_^+^-N content in the soil profile was significantly lower than the nitrate content, and the NH_4_^+^-N content from the different treatments varied (2.6 to 5.2 mg N kg ^−1^) across the soil profile at a depth of 0–200 cm. The soil NH_4_^+^-N content of the GM treatment was slightly lower than that of the FW treatment (Figure S2b).

In the soil layer at 0–200 cm, the FW treatment had the largest accumulation of mineral N (281.7 kg ha^−1^) (Figure 2b). Compared with FW, GM reduced the accumulation of mineral N in the whole soil profile, and the total soil mineral N accumulation (205.8 kg ha^−1^) in the OrV treatment showed the largest reduction (26.9%). Green manuring had a significant impact on the soil mineral N accumulation in the subsoil (40–150 cm). In the soil layer at 40–100 cm, the soil mineral N storage in the RrG and OrV treatments was 76.2 kg ha^−1^ and 51.9 kg ha^−1^, respectively, with a significant decrease of 149.6% and 45.3%. Soil mineral N in the soil layer at a depth of 100–150 cm in the VvR and OrV treatments also decreased significantly by 24.9% and 34.5%, respectively, compared to the FW treatment. In the soil layer at 150–200 cm, there was no significant difference in the accumulation of the soil mineral N within the four treatments (Figure 2b).

**Figure 3:**
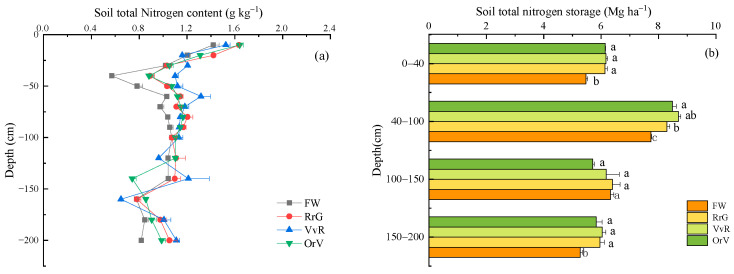
Effects of long-term green manuring on soil total N distribution (**a**) and total N accumulation (**b**) in the soil profile. Error bars are based on standard deviation of three replicates. Different lowercase letters indicate significant differences at the *p*  <  0.05 level within the same treatment group.

Except for the soil mineral N, long-term green manuring also significantly affected the soil total N content of the soil profile (Figure 3a). In the soil layer at a depth of 40–80 cm, the RrG, VvR, and OrV all increased significantly in the total N content compared with the FW, with an average increment of 20.1%, 28.3%, and 19.8%, respectively (Table S5). The total N storage in the 0–200 cm soil profile of the FW, RrG, VvR, and OrV treatment was 24.6, 26.7, 27.1, and 26.2 Mg N ha^−1^, respectively (Figure 3b). The soil total N storage of the GM treatments (RrG, VvR, and OrV) was significantly higher than that of the FW treatment, except for the soil layer at a depth of 100–150 cm.

### 2.4. Nitrification, Denitrification Gene Abundance, N_2_O Production Potential and Correlation Analysis

**Figure 4:**
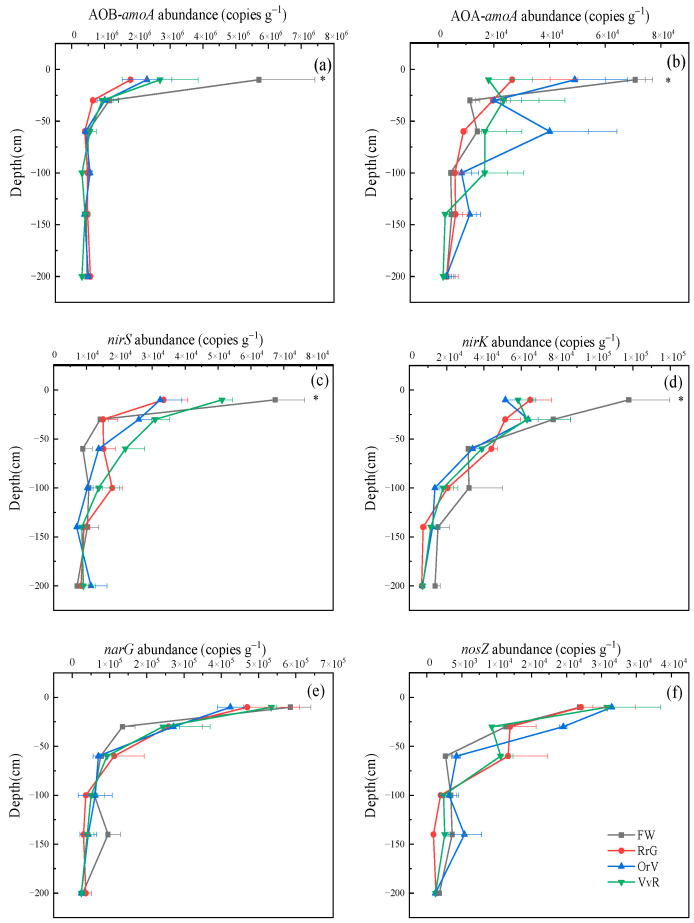
Changes of functional genes (**a**) AOB-amoA, (**b**) AOA-*amoA*, (**c**) *nirK*, (**d**) *nirS*, (**e**) *narG*, and (**f**) *nosZ* in the soil profile. Asterisks indicate that there were significant differences between GM and FW treatment (All three differ from FW) about functional genes analyzed using repeated measures.

To determine the effects of long-term green manuring on soil N cycling, we quantified the abundance of N-cycle genes related to nitrification (AOA-*amoA* and AOB-*amoA*), and denitrification (*nirS, nirK, narG, nosZ,*) (Figure 4). We found a similar pattern in the distribution of *nirS*, *nirK*, *narG*, *nosZ*, AOA-*amoA*, *and* AOB-*amoA* genes (Figure 4a–f) in soil profile, which rapidly reduced with the rise of soil depth. Notably, the AOB-*amoA* (and AOA-*amoA*) abundance of topsoil (0–10 cm) in the RrG, VvR, and OrV treatments significantly decreased by 68.5% (and 60.7%), 52.8% (and 49.7%), and 59.8% (and 59.0%), respectively. Furthermore, in comparison to FW treatment, green manuring reduced the abundance of *nirS*, *nirK* genes at a depth of 0–10 cm, and the abundance of *nirS* (and *nirK*) in RrG, OrV, and VvR treatments significantly decreased by 50.4% (and 44.9%), 51.9% (and 56.2%), and 23.9% (and 50.4%), respectively. There was no significant difference found in *narG* gene between GMs and FW treatment at a depth of 0–10 cm (except for OrV). No significant differences were found in nitrification and denitrification gene abundance for subsoils (>30 cm) (Figure S6).

**Figure 5:**
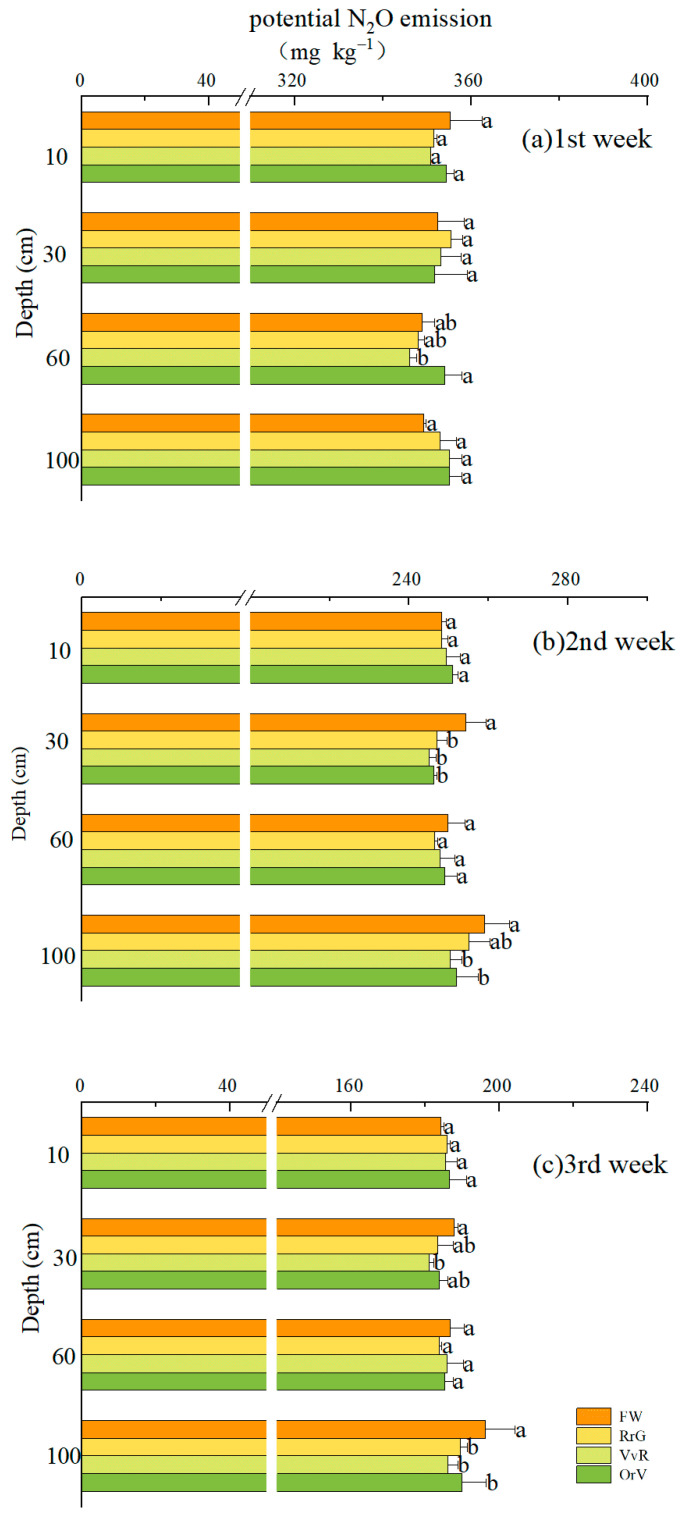
Effects of long-term green manuring on soil N_2_O production potential. Each section represents the cumulative amount of N_2_O seven days. Error bars are based on standard deviation of three replicates. Different lowercase letters indicate significant differences at the *p*  <  0.05 level within the same treatment group.

The first week had the highest cumulative N_2_O production, ranging from 344.5 to 360.4 mg kg^−1^ (Figure 5). Soils were collected from different layers, but most treatments had no significant effect onN_2_O production potential. By the second week and third week, there was a decreasing trend in N_2_O production. Overall, incorporating GMs had no positive impact on the potential to produce N_2_O under laboratory conditions.

**Figure 6:**
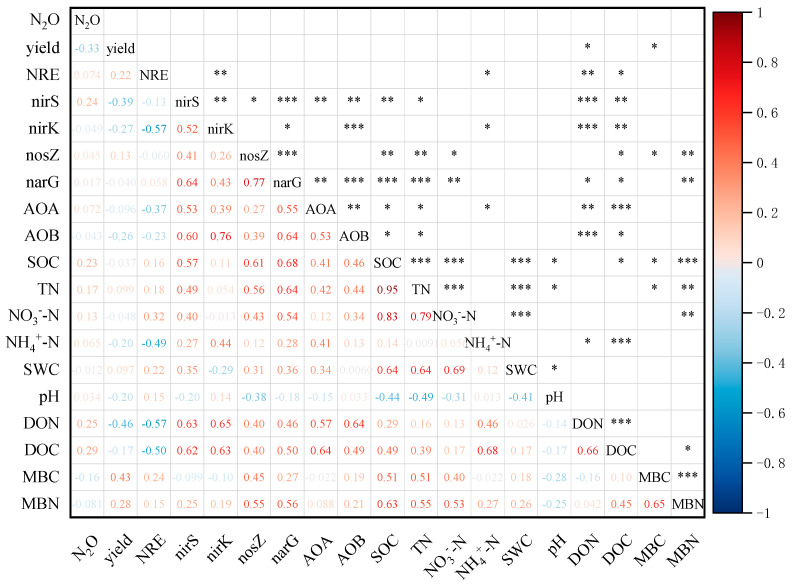
Pearson correlation analysis among the relative abundance of N-cycle functional genes, soil properties, potential N_2_O production, yield and NRE (* *p* ≤ 0.05; ** *p* ≤ 0.01; *** *p* ≤ 0.001. N_2_O, potential N_2_O production; NRE, N recovery efficiency; AOA, archaeal amoA; AOB, bacterial amoA; SOC, soil organic carbon; TN, total nitrogen; NH_4_^+^-N, ammonium; NO_3_^–^-N, nitrate; DON, dissolved organic nitrogen; DOC, dissolved organic carbon; MBN, microbial biomass nitrogen; MBC, microbial biomass carbon).

Pearson’s correlations (Figure 6) revealed that yield was positively correlated with MBC (*p* < 0.05) but negatively correlated to DON (*p* < 0.05). The SOC, TN, DOC, and DON content had positive correlations with majority of N-cycling genes, and MBN only was positively correlated with *narG* and *nosZ* (*p* < 0.01). There was no significantly linear correlation between the N_2_O production potential and the copy numbers of functional genes. The NRE was negatively correlated to *nirK*, NH_4_^−^-N, DOC, and DON. In particular, the NRE showed an extremely negative correlation with DON and *nirK* (*p* < 0.01).

## 3. Discussion

### 3.1. Effects of Long-Term Green Manuring on Crop Yield and N Use Efficiencies

In previous research, green manuring had a significant impact on the yield of subsequent crops, but the results were controversial [26,28]. Some studies suggested that GM has a negative impact on the crop yield, and most of these studies are based on <5 years of field experiment period rather than a long-term field experiment [29]. The results of this study showed that long-term planting and the utilization of GMs had a positive effect on the overall yield of crops. Using GMs for 12 consecutive years resulted in a higher average yield of subsequent crops (RrG > OrV > VvR> FW). The high crop yield was attributed to long-term input of biomass, which promotes the formation of soil organic matter and N cycling and improves the fertility of the soil. The “biological drilling” of GM has been reported to improve the structure of the soil, enhancing the soil water storage and mineral reuse in subsoils [30,31]. Furthermore, GM incorporation can alter microbial community, reduce the relative abundance of potential plant pathogens [32], or improve the abundance of C cycling related functional genes to increased multifunctionality resistance [33]. Therefore, GM improved crop production via enhancing soil fertility and soil health.

Compared with FW, crop N uptake from the treatment planted with VvR (199.8 kg N ha^−1^) was the highest (Table 2), which may be due to the leguminous crops releasing a large amount of mineral N in a short period of time after being incorporated, providing the N nutrient for subsequent crops [34]. The high C/N ratio of non-leguminous crops (RrG and OrV) promoted the fixation of the soil mineral N and enhanced crop N uptake and N use efficiency. Firstly, based on the previous findings, it is likely that the growth of GM converted the active N into organic N (plant biomass), reducing environmental losses and thereby improving crop N uptake and N use efficiency. Secondly, the deep rooting system of non-leguminous GM can uptake the mineral N in deep soil layers. While the current studies show roughly the same total N in FW and GM treatments in topsoil, in previous studies, the deep soil N become available for the subsequent crops after GM being incorporated. GM raised deep soil mineral N from subsoil to the plow layer, improving crop N uptake and N use efficiency [35] (According to previous reports, planting both leguminous and non-leguminous GM can enhance soil nutrients release and crop uptake, thereby improving maize yield and maintaining yield stability [36,37]. Our findings also found that the long-term planting of GM increased N recovery by 6.4–16.7% and fertilizer N partial productivity by 4.6–13.3% compared with FW, and crops have the greatest ability to absorb nitrogen after planting leguminous GM (Table 2).

### 3.2. Effects of Long-Term Green Manuring on Soil Water Storage and pH

Soil water storage is the result of water infiltration, seepage, evapotranspiration, and other water processes, which indicates soil water that is available for plants [38]. Green manuring can reduce the strength of the soil, increase the infiltration rate, and increase soil available water [30,39]. The present study found that long-term planting of GM increased the water content of the soil profile (0–200 cm), which significantly increased the storage of deep soil water (Figure 2). This may be due to (1) the increased surface coverage from planting GM and the reduction in soil evaporation, resulting in an increase in the soil water content [40], and (2) long-term green manuring improves the soil’s water-holding capacity through a decrease in the bulk density and an increase in porosity, enhancing the soil storage in deep layers [41]. This study also found that cruciferous GM (OrV) increased soil water storage the most significantly, which may be due to the relatively deeper root system of the cruciferous family. Overall, GMs can improve soil physical structure by forming bio-pores, which potentially alters the distribution pattern of soil water.

Several studies documented soil pH that either decreased or remained unchanged after planting a GM [42]. In this study, compared with winter fallow, GM treatment decreased the pH in the surface layer of the soil (0–10 cm), which may be due to the formation of carbonic acid from carbon dioxide that is produced by the decomposition of organic matter by microorganisms. This may be related to the incorporation of GMs [31].

### 3.3. Effects of Long-Term Green Manuring on N Distribution in Soil Profile

Non-leguminous GM (*ryegrass*, *Orychophragmus violaceus*, etc.) can absorb part of the soil mineral N and convert it into dry matter during the growth process, thereby reducing the loss of active N in the ecosystem of cropland [21]. Legumes (*hairy vetch*, etc.) can fix N_2_ in the atmosphere, thereby improving the N nutrient in the soil after being incorporated [43]. In this study, the continuous decomposition and mineralization of the winter GM contributed to a higher mineral N content in the soil surface (0–30 cm). The inorganic N in the topsoil was the highest after incorporating Orychophragmus violaceus, followed by ryegrass. Hairy vetch had the smallest amount of mineral N in the topsoil. This may be due to the relatively low C/N ratio of Orychophragmus violaceus and hairy vetch, which decomposes faster than ryegrass [44].

Root depth and its growth rate are key factors in the soil profile of crop N uptake [45]. Previous studies showed that the root depth and density of forage radishes and ryegrass highly correlated with N uptake in the underlying soil. The fine roots of the cruciferous GM of forage radishes can grow to a depth of 2 m. The roots of poaceae ryegrass can grow as deep as one m [46,47]. This study found that, compared with winter fallow, long-term green manuring significantly reduces nitrate in the deep soil by 17.8–39.6%, which are consistent with previous findings [48]. Different GMs demonstrated different effects on removing the subsoil mineral N, following Orychophragmus violaceus > ryegrass > hairy vetch. It may be due to the large root biomass and the deep rooting system of Orychophragmus violaceus and ryegrass (Figure S2) [49]. Legume GMs have relatively shallow root systems, which limit them to the uptake of mineral N from the deep soil. The utilization of subsoil N is not as good as that of Orychophragmus violaceus and ryegrass, but compared with winter fallow, it still reduced the accumulation of the mineral N and the risk of nitrate leaching [43].

This study found that long-term green manuring increased the total N content of the soil (0–80 cm) by 15.8–19.7% compared with winter fallow. The continuous incorporation of GM promoted soil organic matter formation, thereby increasing soil total N [37]. Some studies also reported that root biomass (0.4–0.5 Mg ha^−1^) contributed 2.4 times more soil carbon than the aboveground biomass, which may enhance the subsoil organic N pool [50,51]. In this study, the contribution of the leguminous GM to the soil total N stock was greater than that of the non-leguminous GMs (Orychophragmus violaceus L and ryegrass). The first reason may be that the leguminous GM can fix N_2_ through biological N fixation, and part of the soil organic matter (such as dissolved organic matter) can move to the deep soil, increasing the subsoil organic N stock [52,53]. However, N fixation and diazotrophs were suppressed when legume GM grew in soil that contained high amounts of mineral N [11], especially for annual legumes that have a short period of growing season. Another reason is that the disproportionate increase in N in particulate organic matter fractions promoted the formation of a stable soil organic N pool [54]. Moreover, leguminous GM regulated the N content of cPOM and iPOM in large and small macroaggregates to expand the soil organic N pool [54].

### 3.4. Implication for N_2_O Production

Some studies reported that the labile organic carbon can provide energy for improving soil microbial activity and abundance [55]. In this study, we found the abundance of AOA-*amoA*, AOB-*amoA, nirS* and *nirK* significantly decreased in the GM treatments in the topsoil compared with FW treatment. The difference is probably attribute to the low DOC in GM treatments (Figure S3). Generally, lower C/N and higher NO_3_^−^-N were related to higher N_2_O production potential [56], in the present study, green manuring diminished the quantity of nitrification and denitrification genes to varying degrees for topsoil, which may favor lower nitrification and denitrification rates and the related N_2_O production. There are many possible explanations for this phenomenon: i) nitrous oxide reductase encoded by *nosZ* I and II is more active than the enzymes that produce N_2_O (encoded by *nirS*, *nirK*) [57], or ii) nitrification inhibitors derived from GM root inhibited the activity of related nitrifies [58].

Functional gene is an important factor influencing N_2_O production [59]. Maeda et al. [60] found the N_2_O mainly produced by denitrification and was positively correlated with *nosZ*. Yang et al. [61] documented that AOA was strongly positively correlated with N_2_O. However, we found an unclear link between the related gene abundance with N_2_O production potential. The first reason is that N_2_O production has complex nonlinear and interactions with these functional genes and environmental variables, and Pearson analysis does not demonstrate this nonlinearity and interaction well. For example, enough NO_3_^−^-N (higher N_2_O production potential) and fewer functional genes (lower N_2_O production) made no significant difference to N_2_O production potential in topsoil. The other reason may reflect the limitations of existing molecular approaches [62]. For example, there exists inconsistent change between denitrification genes and N_2_O production at the level of DNA, but they are associated at the mRNA level. N_2_O potential production may also be correlated to environmental factors (enzyme activities and so on) and itself under certain conditions according to previous research [63]. It may also explain the effect of soil physicochemical properties on potential N_2_O production. Several field experiments have been reported that the increase in crop rotation diversity can decrease soil greenhouse gas emissions without sacrificing yields in the NCP [64,65], although we found a reduction in nitrification and denitrification genes at the seedling stage of maize. More samples from different periods are required to reveal the change of functional gene in soil profile. Additionally, N_2_O production needs to be observed directly during the entire crop rotation cycle to understand whether GM as a feasible practice can reduce nutrient loss and N_2_O production in the further study.

## 4. Materials and Methods

### 4.1. Site Description

The winter GM–spring maize rotation experiment began in 2009 in the modern agricultural science and technology innovation base (117°10′ E, 39°21′ N, elevation is 3.6 m) in Wuqing District, Tianjin, China; the site belongs to a warm-temperate and semi-humid continental monsoon climate. The average annual temperature varied from 425.7 to 600.2 mm with an average on the nine years (2013–2021) of 532.5 mm and an annual average temperature of 13.5 °C (Figure S1). The annual sunshine hour and frost-free day is 2810 h 203 days, respectively. The topography of this site is nearly flat, and the fluvo-aquic soil predominates in this study region (Chinese soil classification).

### 4.2. Experimental Design

The experiment was composed of four different rotation systems of winter GM–spring maize; namely, winter fallow–spring maize (FW), ryegrass (*Lolium* L.)–spring maize (RrG), hairy vetch (*Vicia villosa* Roth.)–spring maize (VvR) and Orychophragmus violaceus (*Orychophragmus violaceus* L.)–spring maize (OrV), and the FW mode was additionally set to a non-fertilization treatment (CK) among them, resulting in a total of five treatments. It is well-known that RrG (1.1–1.2 m) and VvR (1.0–2.0 m) have the potential to grow roots to 1 m depth or more [47,66]. The depth of taproot was 0.5–0.8 m in OrV, but it had well-developed lateral roots with the potential to extend to well below 1.0–2.0 m depth [67]. Each treatment was arranged in random blocks with three replicates with a plot size of 3 m × 6 m, with guarding rows. The GMs were sown in mid-to-late September after the spring maize was harvested, and the seeding rates of RrG, VvR, and OrV were 45.0, 60.0, and 60.0 kg ha^−1^, respectively. During the GM growing season, none of the treatments were weeded, fertilized, sprayed with pesticides, or irrigated. In mid-April of the following year, the winter GM was chopped up and incorporated into the topsoil. At the end of April, spring maize was sown at a seeding density of 50,000 plants ∙ha^−1^ with 60 cm of spacing between rows. The fertilization rates of maize were 225.0 kg N ha^−1^, 90.0 kg P_2_O_5_ ha^−1^, and 80.0 kg K_2_O ha^−1^, respectively. Among them, 1/3 of the N fertilizer was applied as a base fertilizer, and the remaining N fertilizer was applied as a top dressing at jointing stage of maize. All phosphate and potassium fertilizers were applied as base fertilizers. The variety of maize used in the field was Zhengdan 958. The types of fertilizers used were urea, diammonium phosphate, and potassium chloride.

### 4.3. Sample Collection and Measurements

#### 4.3.1. Plant Sampling and Analysis

For dry matter of winter GMs, a sample area of 1 × 1 m^2^ was selected for each plot, the aboveground and underground (20 cm) parts of the GM biomass were collected, respectively. Weed samples were taken in the FW treatment. We selected three plants with uniform growth in each plot in which the stalks and grains of the plants were collected when the spring maize matured, the fresh biomass samples were dried at 105 °C for 2 h, and then the dry matter was determined after drying it at 70 °C to a constant weight. The N concentration was determined using the Kjeldahl method following the crushing of the dry plant samples [68]. The yield was calculated by harvesting the spring maize in the entire plot.

#### 4.3.2. Soil Sampling and Analysis

On 26 May 2021, soil samples from 12 soil profiles (0–200 cm) from four different crop rotation patterns were collected after the incorporation of GM. In the soil layer at 0–100 cm, soil samples were collected at an interval of 10 cm. For the soil layer at 100–200 cm, soil samples were collected at an interval of 20 cm. Each soil profile received a total of 15 soil samples. Then, the physicochemical properties were analyzed in the laboratory. The soil water content was measured via the oven-drying method. The pH value of the soil was measured at a water and soil ratio of 5:1. The total nitrogen (TN) and SOC were analyzed via the C/N element analyzer (Flash Smart, Thermo Fisher Scientific, Waltham, MA, USA). The inorganic N concentration (NO_3_^−^-N and NH_4_^+^-N) was measured using the Skalar flow analyzer method (SAN++, Skalar, The Netherlands) after soil samples were extracted using 2 mol L^−1^ KCl. Soil dissolved organic C (DOC) and N (DON) were measured by extraction with ultrapure water at a 1:5 soil/solution ratio. DOC and total dissolved N (DTN) were measured by TOC/N (TOC-L CPH, Shimadzu, Japan), and DON was calculated as difference between DTN and inorganic N (NO_3_^−^-N and NH_4_^+^-N).

#### 4.3.3. Potential N_2_O Production

Potential N_2_O production was measured by quantifying N_2_O release from the incubated soil in 150 mL serum bottles. Each serum bottle, containing the equivalent to 15 g dry soil, was sealed with breathable film to maintain aerobic culture and incubated at 25 °C in a dark incubator. All soils were maintained at a 60% water holding capacity using deionized water, which was changed every two days based on soil weight. Each bottle was sealed with a rubber septum to allow gas sampling from the headspace. Headspace N_2_O releases were measured using a gas chromatogram equipped with flame ionization and electron capture detectors (Agilent 7890A, Santa Clara, CA, USA) at 1, 3, 6, 10, 15, and 21 incubation days. The bottles were opened and flushed with ambient air for 5 min after each sampling.

The rate of gas N_2_O generation is calculated as follows:

(1) $$F=\frac{\rho \times V\times \Delta c}{m\times \Delta t}\times 24$$

(2) $$\rho =\frac{M}{22.4}\times \frac{273}{273+T}$$

where *F* is the gas production rate, (mg kg^−1^ d^−1^); *ρ* is the gas density under standard state; *V* is the effective volume of gas in the culture flask (L); *m* is the dry weight of the soil (g); Δ*t* is the gas production interval (h), 24 for unit conversion; Δ*c* is the change in the concentration of gas in the culture flask (ppm) in Δ*t* time; *T* is the culture temperature (25 °C); *M* is the molecular weight of gas N_2_O (g mol^−1^).

The total amount of N_2_O is calculated as follows:

(3) $$C_{t}{}^{\prime }=C_{t}+\frac{1}{2}\times \left(F_{t}+F_{t}^{\prime }\right)\times \left(t^{\prime }-t\right)$$

where $C_{t}{}^{\prime }$ and $C_{t}$ are the total gas produced at $t^{\prime }$ and t, respectively (mg kg^−1^ d^−1^); $F_{t}^{\prime }$ and $F_{t}$ are the rate at which the gas is produced at *t*’ and *t*, respectively (mg kg^−1^); *t* and *t*’ are sampling time (d) and one sampling time after t (d), respectively.

### 4.4. DNA Extraction and Quantitative Real-Time PCR

Following the manufacturer’s instruction, soil DNA (10 cm, 30 cm, 60 cm, 100 cm, 140 cm, 200 cm) were extracted by Mag-Bind soil DNA Kit (Omega, cat: M5635-02, Radnor, PA, USA), and then we used a UV quantitative device (Nanodrop NC2000, Thermo Scientific, Waltham, MA, USA) and 1.2% agarose gel electrophoresis to determine the concentration and quality of the extracted DNA.

The absolute abundances of functional genes of the N cycle were measured by quantitative polymerase chain reaction (qPCR). The following genes were assessed: ammonia oxidation (AOA-*amoA* and AOB-*amoA*) for nitrification; nitrite reduction (*nirS* and *nirK*), nitrate reduction (*narG*), and nitrous oxide reduction (*nosZ*) for denitrification. The qPCR analyses were performed using Roche LightCycler480 Real-time PCR System with 96-well plates (Axygen, PCR-96-FLT-C, Union City, CA, USA). The primer information can be found in Table S1. Cycle condition was 95 °C for 5 min, followed by 40 cycles of 95 °C for 15 s and 60 °C for 30 s. For all assays, the amplification efficiency ranged between 87% and 96%, and R^2^ values were all greater than 0.99 (Table S1).

### 4.5. Nitrogen Use Efficiencies

Fertilizer N partial factor productivity (NPFP, kg kg^−1^) and N recovery efficiency (NRE, %) can be used to evaluate N use efficiency.

(4) $$NPFP=\frac{Y_{N}}{E_{N}}$$

(5) $$NRE=\frac{A_{N}-A_{0}}{E_{N}}\times 100\%$$

where *Y_N_* is the grain yields (kg ha^−1^) of spring maize in the N fertilized treatment. *E_N_* is the N application of fertilizer (kg N ha^−1^). *A_N_* and *A*_0_ are crop N uptake (kg N ha^−1^) at maturity in the N fertilized and unfertilized treatment, respectively.

### 4.6. Statistical Analysis

IBM SPSS statistics 20 (SPSS Inc. Chicago, IL, USA) was used for data processing and statistical analysis, including normal distribution test (Shapiro–Wilk test and Q-Q plot), homogeneity of variance test (Levene’s test), one-way ANOVA (Duncan), and Pearson correlation analysis. OriginPro 2022b (OriginLab Corporation, Northampton, MA, USA) was used for graphing.

## 5. Conclusions

Results from long-term positioning experiments demonstrated that winter GMs (ryegrass, Orychophragmus violaceus and hairy vetch) are an effective way to increase the yield of spring maize and the N use efficiency in NCP. Compared with the winter fallow, after 10 years of green manuring, the spring maize yield of ryegrass and Orychophragmus violaceus significantly increased by 6.3–14.7%, 11.5–16.6%, respectively. Winter GM increased soil water storage by 4.9–17.1% and significantly reduced subsoil (80–120 cm) nitrate, especially for the deep-rooted GMs (ryegrass and Orychophragmus violaceus), demonstrating positive recovery of deep soil N. Long-term winter green manuring also increased the total N content in the soil layer at a depth of 0–80 cm by 15.8–19.7%, and the total N stock of the leguminous GM treatment (13.5 Mg ha^−1^) was significantly higher than the other treatments. In addition, winter green manuring effectively reused mineral N in the deep soil layer and improved N use efficiency. The N recovery rate and fertilizer N partial productivity increased by 10.4–32.7% and 4.6–13.3%, respectively. This study suggested that the winter GM rotation strategy increased crop yield and N recovery efficiency in NCP.

## Figures and Tables

**Table 1:** Effects of different GMs on the spring maize yield from 2013 to 2020 (kg ha^−1^).

Year	FW	RrG	VvR	OrV
2013	7517 a	7057 a	5793 a	6231 a
2014	9502 a	10,143 a	10,110 a	9810 a
2015	7503 a	7963 a	7377 a	6780 a
2016	8204 a	9037 a	8145 a	8420 a
2017	8205 a	8555 a	8580 a	8421 a
2018	7448 a	8510 a	8888 a	8226 a
2019	8086 c	8599 b	9030 ab	9434 a
2020	8426 b	9662 a	8515 b	9395 a

Different lowercase letters indicate significant differences among different GMs (*n* = 3, *p* < 0.05).

**Table 2:** Effects of different winter GMs on N uptake and N use efficiencies of maize.

Treatments	Maize Grain	Maize Straw	N Uptake	NPFP	NRE
	(kg N ha^−1^)	(kg N ha^−1^)	(kg N ha^−1^)	(kg kg^−1^)	(%)
FW	93.1 c	74.1 b	167.2 b	36.9 b	44.4 b
RrG	108.8 b	85.1 a	193.9 a	40.6 a	56.3 a
VvR	129.3 a	70.4 b	199.8 a	38.6 a	58.9 a
OrV	114.8 b	62.6 b	177.4 b	41.8 a	49.0 a

Different lowercase letters indicate significant differences among different GMs (*n* = 3, *p* < 0.05), NPFP is fertilizer N partial factor productivity, NRE is N recovery efficiency.

## Data Availability

The datasets generated for this study are available on request to the corresponding author.

## Supplementary Materials

The following supporting information can be downloaded at: https://www.mdpi.com/article/10.3390/plants12020311/s1, Table S1: Primer pairs and PCR conditions for quantitative PCR; Table S2: Dry matter yield of different winter cover crops on 2021; Table S3: Effects of long-term cover crops on soil moisture in the soil profile; Table S4 Effects of long-term cover crops on nitrate content in the soil profile.; Table S5: Effects of long-term cover crops on total nitrogen content in the soil profile; Table S6: Abundances of AOB-amoA, AOA-amoA, nirS, narG nirK, nosZ genes in the soils under different cover crop treatments of fallow-spring corn (FW), ryegrass-spring corn (RrG), orychophragmus violaceus-spring corn (OrV) and hairy vetch-spring corn (VvR). Figure S1: Mean monthly temperature and cumulative precipitation from January 2013 to May 2021 at the experimental site; Figure S2: Effects of long-term cover crops on soil pH and ammonia-N content in the soil profile; Figure S3: Effects of long-term cover crops on soil dissolved organic carbon in the soil profile.
