# Supplementary material for: Winter Green Manure Decreases Subsoil Nitrate Accumulation and Increases N Use Efficiencies of Maize Production in North China Plain
Source: Plants (Basel). 2023 Jan 9;12(2):311. doi: 10.3390/plants12020311 (PMC9866620; doi:10.3390/plants12020311)
Supplement: Supplementary file 1 [file plants-12-00311-s001.zip › plants-2096288-supplementary.pdf]

Table S1. Primer pairs and PCR conditions for quantitative PCR

Target genes	Primer	Sequence (5’-3’)	Amplification efficiency ($R2 > 0.99$) (%)	References
AOA-*amoA*	Arch-amoA26F	STAATGGTCTGGCTTAGACG	96.10	(Wang et al., 2019)
	Arch-amoA417R	GCGGCCATCCATCTGTATGT		
AOB-*amoA*	amoB-1F	GGGGTTTCTACTGGTGGT	94.90	(Wang et al., 2019)
	amoB-2R	CCCCTCKGSAAAGCCTTCTTC		
*nir*S	cd3aF	GTSAACGTSAAGGARACSGG	92.35	(Shi et al., 2019)
	R3cdR	GASTTCGGRTGSGTCTTGA		
*nir*K	nirKF1aCu	ATCATGGTSCTGCCGCG	92.70	(Shi et al., 2019)
	nirKR3Cu	GCCTCGATCAGRTTGTGGTT		
narG	narG-1960m2f	TAYGTSGGGCAGGARAAACTG	91.64	(Di et al., 2014)
	narG-2050m2r	CGTAGAAGAAGCTGGTGCTGTT		
*nos*Z	nosZ-1126F	GGGCTBGGGCCRTTGCA	87.60	(Wang et al., 2022)
	nosZ-1381R	GAAGCGRTCCTTSGARAACTTG		

Table S2. Dry matter yield of different winter cover crops on 2021

Treatment	Aboveground biomass （kg ha^{-1}）	Undergroundbiomass（kg ha^{-1}）	Total biomass （kg ha^{-1}）
FW	1050±16.51c	263±3.68c	1312±11.02c
RrG	3301±19.67b	2250±31.56a	5551±18.84b
OrV	4752±27.25a	1900±13.66a	6653±32.13a
VvR	3801±23.44b	1190±21.94b	4991±10.35b

Note: Different lowercase letters indicate significant differences among different cover crops (n = 3, $P < 0.05$).

Table S3. Effects of long-term cover crops on soil moisture in the soil profile.

Depth(cm)	FW	RrG	VvR	OrV
10	0.33±0.02b	0.39±0.00a	0.32±0.02b	0.42±0.01a
20	0.34±0.01b	0.36±0.01b	0.37±0.02ab	0.40±0.00a
30	0.33±0.01b	0.37±0.01a	0.33±0.01b	0.39±0.00a
40	0.33±0.01b	0.36±0.01ab	0.33±0.02b	0.39±0.01a
50	0.30±0.00c	0.34±0.01b	0.33±0.02bc	0.40±0.01a
60	0.30±0.00b	0.31±0.01b	0.31±0.02b	0.36±0.01a
70	0.29±0.00b	0.29±0.01b	0.25±0.00c	0.35±0.01a
80	0.26±0.01c	0.27±0.01bc	0.29±0.01b	0.34±0.01a
90	0.23±0.00b	0.26±0.01b	0.24±0.00b	0.33±0.00a
100	0.24±0.01b	0.29±0.01a	0.28±0.01a	0.29±0.01a
120	0.23±0.01b	0.25±0.01b	0.23±0.01b	0.30±0.01a
140	0.16±0.00b	0.25±0.01a	0.27±0.01a	0.28±0.00a
160	0.18±0.01a	0.19±0.01a	0.19±0.02a	0.20±0.01a
180	0.20±0.03b	0.19±0.01b	0.26±0.01a	0.22±0.02b
200	0.22±0.04b	0.24±0.01ab	0.26±0.01a	0.25±0.03ab

Results are reported as means ± SD (n = 3). Different letters within a row indicate significant differences between the treatments by LSD-Test least significant difference (p<0.05).

Table S4. Effects of long-term cover crops on NO_3^--N content in the soil profile.

	Depth (cm)	FW	RrG	VvR	OrV
NO_3^--N	10	14.51±1.46c	21.11±3.49b	27.66±1.92a	15.69±1.71c
	20	6.83±1.16c	16.17±0.42a	10.79±0.26b	10.54±0.66b
	30	5.83±1.12a	6.02±1.64a	6.80±0.53a	6.33±1.31a
	40	4.85±0.49a	5.98±1.01a	5.99±0.53a	5.15±0.55a
	50	6.18±1.00a	6.68±1.28a	7.29±0.63a	4.74±0.40a
	60	7.84±1.52a	7.20±1.02a	7.61±0.80a	6.04±0.29a
	70	7.60±1.09ab	7.89±1.64ab	9.46±1.12a	5.60±0.74b
	80	8.77±0.31a	7.91±0.24a	7.30±0.59ab	5.10±0.07b
	90	8.27±0.36a	6.50±1.00a	7.07±1.00a	5.66±0.30a
	100	9.14±0.28a	5.25±0.85b	6.94±0.68ab	6.74±1.44ab
	120	7.82±0.53a	5.37±0.90ab	6.62±0.50ab	4.77±0.06b
	140	6.45±0.79a	6.39±0.40a	5.04±0.21a	3.87±0.40a
	160	5.63±0.51a	4.14±0.13a	4.38±0.16a	5.93±1.87a
	180	3.91±0.35a	3.15±0.12a	4.83±1.10a	4.96±1.51a
	200	3.48±0.23a	2.74±0.14a	3.70±0.63a	3.53±0.39a

Results are reported as means ± SD (n = 3). Different letters within a row indicate significant differences between the treatments by LSD-Test least significant difference (p<0.05).

Table S5. Effects of long-term cover crops on total nitrogen content in the soil profile.

	Depth(cm)	FW	RrG	VvR	OrV
	10	1.42±0.05b	1.63±0.01a	1.52±0.04ab	1.64±0.03a
	20	1.20±0.02bc	1.42±0.01a	1.16±0.05c	1.31±0.01ab
	30	1.02±0.03b	1.03±0.04b	1.21±0.01a	1.05±0.03b
	40	0.57±0.00c	0.89±0.04b	1.10±0.03a	0.88±0.04b
	50	0.78±0.05b	1.03±0.02a	1.12±0.05a	1.07±0.00a
TN	60	1.03±0.02b	1.14±0.02b	1.32±0.08a	1.12±0.04b
	70	0.98±0.03b	1.11±0.05a	1.18±0.03a	1.15±0.05a
	80	1.04±0.01b	1.20±0.05a	1.14±0.03ab	1.17±0.01a
	90	1.06±0.03a	1.17±0.02a	1.13±0.01a	1.14±0.01a
	100	1.07±0.02a	1.07±0.04a	1.13±0.03a	1.10±0.03a
	120	1.04±0.00ab	1.11±0.08a	0.96±0.02b	1.10±0.02a
	140	1.04±0.02b	1.10±0.05ab	1.21±0.18a	0.74±0.03c
	160	0.78±0.01a	0.78±0.03a	0.65±0.00b	0.86±0.01a
	180	0.85±0.03b	0.98±0.05a	1.01±0.06a	0.91±0.04ab
	200	0.82±0.01c	1.05±0.06ab	1.11±0.03a	0.99±0.03b

Results are reported as means ± SD (n = 3). Different letters within a row indicate significant differences between the treatments by LSD-Test least significant difference ($p<0.05$).

Table S6. Abundances of AOB-*amoA*, AOA-*amoA, nirS, narG*　*nirK*, *nosZ* genes in the soils under different cover crop treatments of fallow-spring corn (FW), ryegrass-spring corn (RrG)，orychophragmus violaceus-spring corn (OrV) and hairy vetch-spring corn（VvR）.

		**Gene copy numbers (copies/g dry soil)**					
Depth(cm)	Treatment	AOB-*amoA*	AOA-*amoA*	*nirS*	*nirK*	*narG*	*nosZ*
10	FW	4566267±3563647a	4566267±3563647a	76348±26833a	117758±38023a	584964±96565a	21856±3324a
	RrG	1794492±870235b	1794492±870235b	33426±12855c	67891±20598b	469600±243148ab	22068±13421a
	OrV	2294648±1311140b	2294648±1311140b	32402±11395c	51535±28390b	424309±60621b	26464±265a
	VvR	2694243±2012945b	2694243±2012945b	51233±5430b	54439±16137b	534524±23481ab	25794±13253a
30	FW	955869±598919a	955869±598919a	14191±4102b	77243±15888a	135067±57539a	11300±1058b
	RrG	644091±166994a	644091±166994a	15037±7404b	51419±14395b	258683±156655a	11912±6487ab
	OrV	1011870±422380a	1011870±422380a	25878±6838ab	63776±9138ab	271510±27638a	19527±943a
	VvR	930769±840097a	930769±840097a	30906±7569a	39895±6673b	243358±219860a	9304±3186b
	FW	484371±110531a	484371±110531a	8880±5055a	31579±14490a	76210±52071a	2671±1507b

60	RrG	401260±100005a	401260±100005a	15174±6287a	43995±5916a	112795±139280a	11618±9880a
	OrV	423383±52917a	423383±52917a	13706±2724a	33897±9821a	69980±24380a	4256±1059ab
	VvR	554498±342966a	554498±342966a	21812±10144a	38730±1251a	93103±37887a	10554±3048ab
100	FW	517157±171947a	517157±171947a	10699±7248a	31988±31354a	59175±48535a	3430±1396a
	RrG	482449±30879a	482449±30879a	17829±5200a	20580±5089a	36337±11581a	1966±1119a
	OrV	559911±116193a	559911±116193a	10258±3058a	13696±3712a	61625±79129a	3187±2356a
	VvR	314986±79846a	314986±79846a	13695±10983a	18114±13753a	50501±31393a	2380±2093a
140	FW	443813±3045a	443813±3045a	10284±5913a	15360±10431a	95976±57409a	3628±3323a
	RrG	487125±39377a	487125±39377a	9204±3188a	7362±2947a	30330±23212a	948±452a
	OrV	390339±97088a	390339±97088a	7009±3400a	12483±4086a	42977±39429a	5377±4287a
	VvR	405941±258960a	405941±258960a	8570±2833a	11514±7262a	39948±30447a	2561±1000a
200	FW	468786±46545a	468786±46545a	7117±1333a	13765±4706a	27951±13730a	1802±127a
	RrG	569267±48980a	569267±48980a	8358±3807a	6625±1140a	36208±26087a	1314±390a
	OrV	508399±126247a	508399±126247a	11328±8483a	6970±1857a	24420±9778a	1226±274a
	VvR	313049±100640a	313049±100640a	9047±6393a	7126±1859a	24281±14380a	1237±110a

Note: Results are reported as means ± SD (n = 3). Different letters within a row indicate significant differences between the treatments by LSD-Test least significant difference ($P<0.05$).

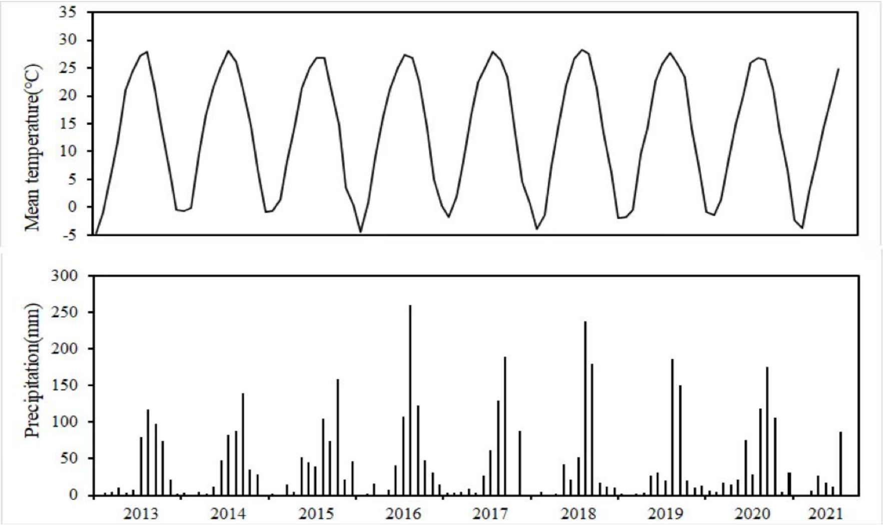

Figure S1. Mean monthly temperature and cumulative precipitation from January 2013 to May 2021 at the experimental site.

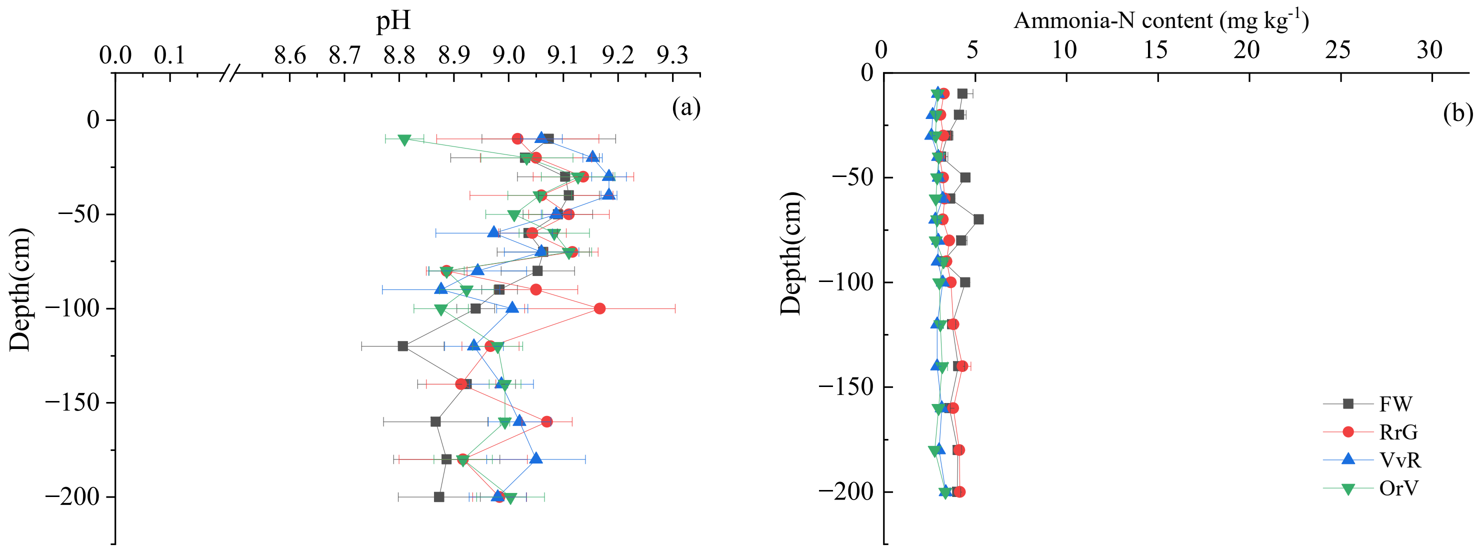

Figure S2. Effects of long-term cover crops on soil pH and ammonia-N content in the soil profile.

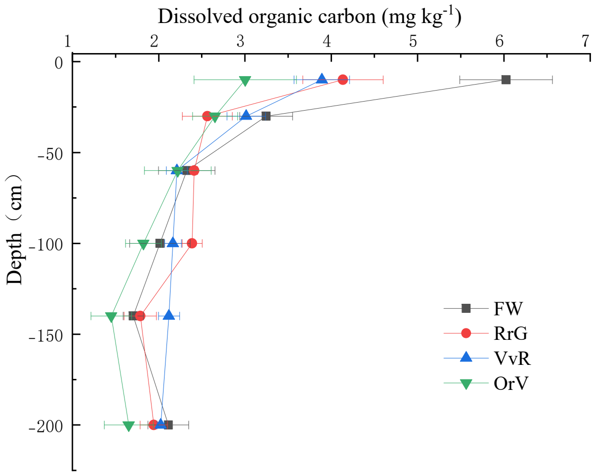

Figure S3. Effects of long-term cover crops on soil dissolved organic carbon in the soil profile.

References (not listed in the main text)

Di, H.J., Cameron, K.C., Podolyan, A., Robinson, A., 2014. Effect of soil moisture status and a nitrification inhibitor, dicyandiamide, on ammonia oxidizer and denitrifier growth and nitrous oxide emissions in a grassland soil. Soil Biology and Biochemistry 73, 59-68.

Shi, Y., Liu, X., Zhang, Q., 2019. Effects of combined biochar and organic fertilizer on nitrous oxide fluxes and the related nitrifier and denitrifier communities in a saline-alkali soil. Science of the Total Environment 686, 199-211.

Wang, J., Wang, J., Rhodes, G., He, J., Ge, Y., 2019. Adaptive responses of comammox Nitrospira and canonical ammonia oxidizers to long-term fertilizations: Implications for the relative contributions of different ammonia oxidizers to soil nitrogen cycling. Science of the Total Environment 668, 224-233.

Wang, L., Xu, H., Liu, C., Yang, M., Zhong, J., Wang, W., Li, Z., Li, K., 2022. Stronger link *of nosZI* than *nosZII* to the higher total N2O consumption in anoxic paddy surface soils. Geoderma 425, 116035.
